# Circulating 25-Hydroxyvitamin D and 1,25-Dihydroxyvitamin D Concentrations and Postoperative Infections in Cardiac Surgical Patients: The CALCITOP-Study

**DOI:** 10.1371/journal.pone.0158532

**Published:** 2016-06-29

**Authors:** Armin Zittermann, Joachim Kuhn, Jana B. Ernst, Tobias Becker, Julia Larisch, Jens Dreier, Cornelius Knabbe, Jochen Börgermann, Jan F. Gummert

**Affiliations:** 1 Clinic for Thoracic and Cardiovascular Surgery, Heart and Diabetes Center North Rhine-Westphalia, Ruhr University Bochum, Bad Oeynhausen, Germany; 2 Institute for Laboratory and Transfusion Medicine, Heart and Diabetes Center North Rhine-Westphalia, Ruhr University Bochum, Bad Oeynhausen, Germany; University of Alabama at Birmingham, UNITED STATES

## Abstract

**Background:**

Vitamin D has immunomodulatory properties and seems to reduce the risk of infections. Whether low vitamin D concentrations are independent risk factors for nosocomial postoperative infections in surgical patients remains to be studied in detail.

**Methods:**

In 3,340 consecutive cardiac surgical patients, we investigated the association of circulating 25-hydroxyvitamin D (25OHD; indicator of nutritional vitamin D status) and 1,25-dihydroxyvitamin D (1,25[OH]_2_D; active vitamin D hormone) with nosocomicial infections. The primary endpoint was a composite of thoracic wound infection, sepsis, and broncho-pulmonary infection. Vitamin D status was measured on the last preoperative day. Infections were assessed until discharge. Logistic regression analysis was used to examine the association between vitamin D metabolite concentrations and the composite endpoint.

**Results:**

The primary endpoint was reached by 5.6% (n = 186). In patients who reached and did not reach the endpoint, in-hospital mortality was 13.4% and 1.5%, respectively (P<0.001). Median (IQR) 25OHD and 1,25(OH)_2_D concentrations were 43. 2 (29.7–61.9) nmol/l and 58.0 (38.5–77.5) pmol/l, respectively. Compared with the highest 1,25(OH)_2_D quintile (>81.0 pmol/l), the multivariable–adjusted odds ratio of infection was 2.57 (95%CI:1.47–4.49) for the lowest 1,25(OH)_2_D quintile (<31.5 pmol/l) and 1.85 (95%CI:1.05–3.25) for the second lowest quintile (31.5–49.0 pmol/l). There was no significant association between 25OHD concentrations and the primary endpoint.

**Conclusions:**

Our data indicate an independent association of low 1,25(OH)_2_D levels with the risk of postoperative infections in cardiac surgical patients. Future studies should pay more attention on the clinical relevance of circulating 1,25(OH)_2_D and its regulation.

## Introduction

Nosocomial infections occur worldwide and are a significant burden both for the patients and for public health [1]. Infections of surgical wounds, the urinary tract, and the lower respiratory tract are the most frequent infections [1]. Cardiac surgical patients are at an increased risk of developing nosocomial infections. Incidence rates of up to 20% and more have been reported [2]. Although serious postoperative complications are uncommon, they are potentially devastating. For example, in a large study in 331,429 cardiac surgical patients, patients who developed an infection were more likely to have a prolonged in-hospital stay compared with patients who did not develop a postoperative infection [3]. In addition, in-hospital mortality was significantly higher in patients with major postoperative infection (17.3% versus 3.0%).

Antibiotic prophylaxis has demonstrated a large benefit in the prevention of wound infection [4] and is therefore standard practice in cardiac surgery. Cardiac-surgery-specific guidelines advocate antibiotic prophylaxis for up to 48 h post-operatively [5,6]. Standard or double-dose cefazolin or cefuroxime are most commonly recommended [5,7–11]. However, antibiotic prophylaxis does not completely prevent nosocomial infections [12].

Besides its pivotal role in musculoskeletal health, vitamin D has a broad range of additional effects, which also cover the immune system [13]. There is likely evidence that vitamin D decreases the risk of airway infections [14,15]. Moreover, vitamin D supplements seem to decrease the need for antibiotics [16], especially in elderly patients [17]. In cardiac surgical patients, the prevalence of vitamin D deficiency (circulating 25-hydroxyvitamin D [25OHD] < 30 nmol/l) is between 15% and 38% [18–20].

The present study aimed to investigate whether low vitamin D status is associated with an increased risk of developing postoperative infections.

## Methods

### Patients

This investigation is based on data of the CALCITOP (**Ca**lcitrio**l** and **c**lin**i**cal ou**t**c**o**me in cardiac surgical **p**atients) study, a prospective cohort study of 3,852 cardiac surgical patients [21]. Study participants were recruited between February 2012 and December 2013 at a tertiary heart center in East-Westphalia, Germany (Heart and Diabetes Center North Rhine-Westphalia, Bad Oeynhausen). Eligible for inclusion were cardiac surgical patients aged 18 years and over. Patients with heart transplants and pacemaker/defibrillator implants were excluded. In 3,340 patients, preoperative serum concentrations of 25OHD and 1,25(OH)_2_D as well as postoperative data on infections were available. This dataset was used to perform the statistical analyses. The study was approved by the Ethics Committee of the Ruhr University Bochum at Bad Oeynhausen and was registered at clinicaltrials.gov as NCT02192528. All patients provided their written informed consent.

### Data collection

We prospectively collected preoperative, perioperative, and postoperative data using the electronic patient databases of our institution. Besides 25OHD (indicator of nutritional vitamin D status) and 1,25(OH)_2_D (active, hormonal form of vitamin D), we assessed additional preoperative and surgical characteristics such as age, gender, body mass index (BMI), left ventricular ejection fraction (LVEF), current smoking, diabetes mellitus, concentrations of creatinine, glucose, C-reactive protein (CRP) and leukocytes, previous cardiac surgery, operation priority, and type of surgery. Moreover, we assessed four postoperative outcome parameters, namely infection, intensive care unit (ICU) stay, in-hospital stay, and in-hospital mortality.

### Antibiotic Prophylaxis and Treatment

According to institutional standards, antibiotic prophylaxis was performed routinely with 2g cephalosporin (Cefazolin-Sandoz, Sandoz Pharmaceuticals, Basel Switzerland), preoperatively, intra-operatively and during postoperative drainage (up to 48 h after surgery). In case of an infection, antibiotic therapy was selected according to the sensitivity of the organism and the clinical response of the patient.

### Biochemical Analyses

Circulating levels of 25OHD and 1,25(OH)_2_D, creatinine, CRP, leukocytes, and glucose levels were measured as previously described [21]. Estimated glomerular filtration rate (eGFR) was calculated using the creatinine-based modification of diet in renal disease formula. Routine microbiological tests were used to assess the type of pathogen in case of an infection.

### Endpoints

The primary endpoint was a composite of clinically relevant infections such as thoracic wound infection, sepsis, and broncho-pulmonal infection. Infections were diagnosed according to standard procedures, such as the presence of positive results of microbial culture, pyrexia, tachycardia, and tachypnea. Events were assessed until discharge. Additional endpoints were urogenital and other infections, ICU stay, in-hospital stay, and in-hospital mortality.

### Statistics

Categorical variables are summarized as percentages. Since several continuous variables were non-normally distributed (CRP, glucose), continuous variables are reported as median and interquartile range (IQR). We used the Kolmogorov-Smirnov test to check normal data distribution of continuous variables. Normal distribution was a consideration when probability values were >0.05. Differences in preoperative categorical variables and continuous variables between the groups with and without infection were assessed using Fisher’s exact test and the Mann-Whitney U-test, respectively.

We carried out multiple logistic regression analysis to assess the independent relationship of the preoperative 25OHD or 1,25(OH)_2_D_3_ categories with the risk of infection. According to previous classification [21], we used the following cut-off values for classifying 25OHD: risk of deficiency (<30 nmol/L), risk of inadequacy (30 nmol/L to 49.9 nmol/L), borderline status (50–74.9 nmol/L), adequacy (75–100 nmol/L), and potentially harmful (>100 nmol/L, to convert nanomolar to nanogram per milliliter divide by 2.496). The group with adequate vitamin D status was used as the reference group. Regarding 1,25(OH)_2_D, we divided the study cohort into quintiles, since no generally accepted classification exists. We performed age- and gender-adjusted analyses and used multivariable-adjusted models to examine the association between vitamin D metabolites and the incidence of the primary endpoint. Since the number of variables that can be included for multivariable testing is equal to the square root of the number of events [22], we restricted the covariates to important demographic parameters (age, gender, and BMI), cardiac surgical-related parameters (redo, operation priority, type of cardiac surgery), and additional parameters that differed significantly between patients who reached or did not reach the primary endpoint (see Table 1). We calculated absolute (incidence) rates and odds ratios (ORs) and corresponding 95% confidence intervals (CI). In subgroup analyses, we restricted the study cohort to non-diabetes patients and patients aged ≥ 70 years. In sensitivity analyses, we used 50 nmol/l as cutoff for the lowest 25OHD category and divided the study cohort into tertiles of 1,25(OH)_2_D levels. Moreover, we compared the status of vitamin D metabolites in patients with any infection (broncho-pulmonal infection, thoracic wound infection, sepsis, urogenital infection or other infection) with non-infected patients. To reduce the risk of study bias through already existing preoperative infections, we also restricted the analysis to a comparison between patients with thoracic wound infection and patients without infection. We considered P values <0.05 as statistically significant. All P values are reported two-sided. Analyses were performed using the statistical software package IBM® SPSS®, version 21.

**Table 1:** Baseline characteristics of patients with and without wound infection.

Parameter	Without Infection n = 3154	With Infection n = 186	P value
Age (years)	71 (62–77)	73 (63–78)	0.086
Gender, Males (%)	66.9	67.2	>0.999
Body Mass Index (kg/m^2^)	27.1 (24.6;30.1)	27.4 (24.7;31.4)	0.492
Left-Ventricular Ejection Fraction (%)	60 (51–65)	56 (48–62)	<0.001
EuroSCORE (logistic)	5.1 (2.4;10.9)	8.4 (3.7;19.9)	<0.001
Smoker (%)	28.5	30.6	0.713
Diabetes Mellitus (%)	25.9	40.3	<0.001
Re-Do (%)	9.7	23.7	<0.001
NYHA Class > II (%)	41.0	55.9	<0.001
Operation Priority, Urgent/Emergent (%)	3.9	14.0	<0.001
eGFR (ml/min/1.73m^2^)	77.4 (61.5;90.2)	68.9 (49.5;89.1)	0.001
C-Reactive Protein (mg/l)	2.5 (1.1;6.6)	7.5 (2.5;26.0)	<0.001
Glucose (mg/dl)	105 (94;129)	111 (92;138)	0.384
Type of Surgery			
CABG (%)	35.7	28.5	0.048
Valve Surgery (%)	36.5	39.6	0.059
Combined CABG and Valve Surgery (%)	15.1	23.1	0.005
Others (%)	12.7	18.8	0.024
On-Pump Surgery (%)	66.9	68.3	0.749

CABG: coronary artery bypass graft

## Results

The incidence of the primary endpoint was 5.6% (n = 186). In detail, broncho-pulmonary infection was most prevalent (n = 112), followed by thoracic wound infection (n = 76), including 13 patients with deep sternal wound infection, and sepsis (n = 19). In some patients (n = 21) more than one infection was diagnosed. The incidence of urinary tract and other infections requiring antibiotic treatment like veneous catheter infection, foot infection, and oto-laryngeal or anal smear-positive antibiotic-resistant pathogens was 1.6% (n = 52) and 11.1% (n = 372), respectively. No postoperative case of endocarditis appeared. Patients who reached the primary endpoint were infected with different types of bacteria, among them Staphylococcus epidermidis (n = 51) and other coagulase-negative Staphylococci (n = 44), Enterococci faecalis and faecium (n = 37), Serratia marcescens (n = 18), Escherichia coli (n = 12), Staphylococcus aureus (n = 11), Pseudomonas aeruginosa (n = 10), Enterobacter cloacae (n = 9), and others (n = 51). Moreover, some patients were infected with Candida albicans (n = 78), non-albicans Candida species (n = 48), and Aspergillus fumigatus (n = 4).

Patients who reached the primary endpoint differed significantly from other patients concerning various demographic parameters, several preoperative clinical variables, and type of surgery (Table 1).

However, gender distribution, BMI, smoking status, blood glucose levels, and percent on-pump surgery did not differ significantly between groups.

Median (IQR) 25OHD and 1,25(OH)_2_D concentrations were 43.2 (29.7–61.9) nmol/l and 58.0 (38.5–77.5) pmol/l, respectively. Circulating preoperative 1,25(OH)_2_D levels, but not circulating 25OHD levels, were significantly lower in patients who reached the primary endpoint than in other patients (Table 2).

**Table 2:** Vitamin D metabolites and postoperative outcomes in patients with and without wound infection.

Parameter	Without Infection n = 3154	With Infection n = 186	P value
Preoperative Vitamin D Metabolites			
25-hydroxyvitamin D (nmol/l)	43.2 (30.0;61.9)	42.1 (30.0;60.4)	0.235
1,25-dihydroxyvitamin D (pmol/l)	58.5 (39.5;78.0)	42.5 (24.5;65.8)	<0.001
Mechanical Ventilator Support (h)	9 (7;13)	105 (12;414)	<0.001
Intensive-Care Unit stay (h)	24 (20;66)	240 (44;668)	<0.001
In-Hospital Stay (days)	13 (11;15)	21 (15;40)	<0.001
In-Hospital Mortality (%)	1.5	13.4	<0.001

Continuous data are presented as median with interquartile range

**Fig 1:**
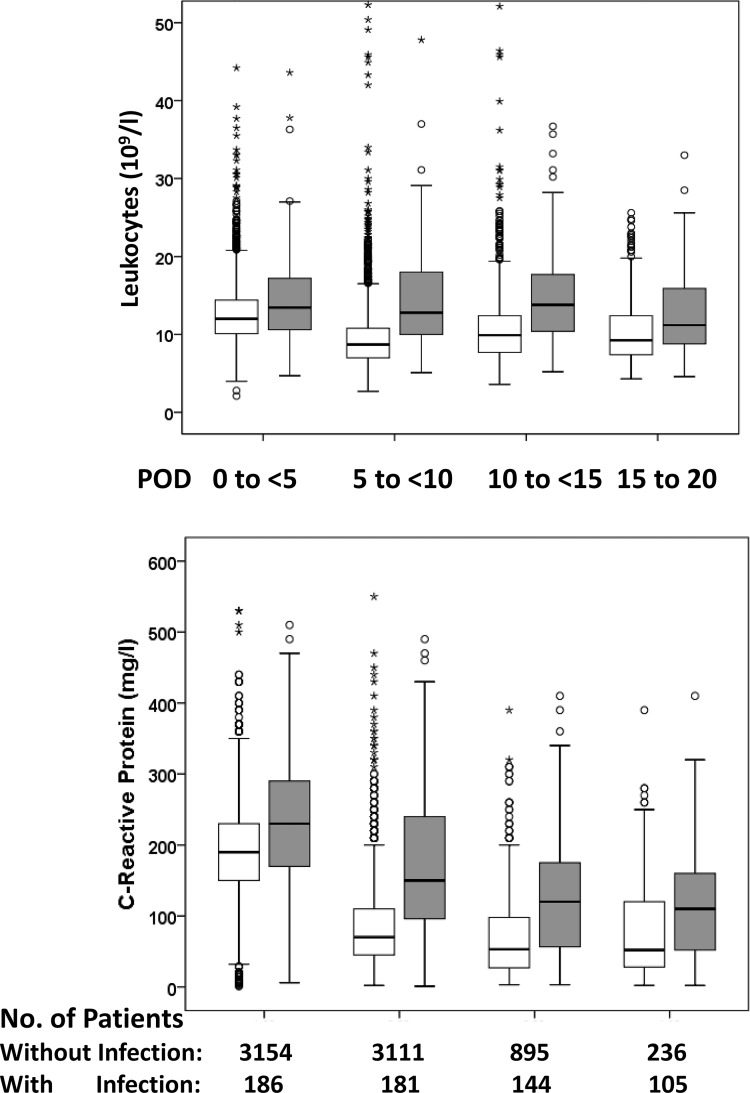
Postoperative time course of leukocyte and C-reactive protein concentrations in cardiac surgical patients with and without infection. The boxes express the upper and lower quartiles, and the central lines show the median. The whiskers represent the values below and above the interquartiles. The circles illustrate outliers, and the stars denote extremes. white boxes, patients without infection; grey boxes, patients with infection; POD, postoperative day

During postoperative hospitalization, infected patients had higher median leukocyte and CRP concentrations than patients who did not reach the primary endpoint (Fig 1).

Moreover, ICU-stay and in–hospital stay were longer, and in-hospital mortality was significantly higher in infected patients than in other patients (13.4% vs. 1.5%, Table 2). Of the 25 patients who reached the primary endpoint and died, causes of death were sepsis (n = 8), multiorgan failure (n = 6), heart failure (n = 6), cardiogenic shock (n = 1), and others (n = 4).

Of the study cohort, 25.5% had deficient 25OHD levels (< 30 nmol/l). Patients in the lowest 1,25(OH)_2_D quintile had 1,25(OH)_2_D levels < 31.5 pmol/l. In Table 3, the ORs for the primary endpoint are given by categories of 25OHD and 1,25(OH)_2_D.

**Table 3:** Unadjusted and adjusted odds ratio (OR) for wound infection by cutoffs of 25-Hydroxyvitamin D and 1,25-Dihydroxyvitamin D.

Vitamin D	N	Primary Endpoint N (%)	Model 1 OR (95% CI)	Model 2 OR (95% CI)	Model 3 OR (95% CI)	Model 4 OR (95% CI)
**25OHD**						
<30 nmol/l	838	58 (6.9)	1.77 (0.96–3.28)	1.94 (1.03–3.65)	1.83 (0.97–3.47)	1.62 (0.87–3.20)
30–49.9 nmol/l	1183	56 (4.7)	1.15 (0.62–2.14)	1.28 (0.68–4.41)	1.30 (0.69–2.46)	1.28 (0.67–2.43)
50–74.9 nmol/l	850	47 (5.5)	1.36 (0.72–2.55)	1.54 (0.81–2.93)	1.60 (0.84–3.07)	1.56 (0.81–3.02)
75–100 nmol/l	318	13 (4.1)	1.0 (reference)	1.0 (reference)	1.0 (reference)	1.0 (reference)
>100 nmol/l	151	12 (7.9)	2.01 (0.72–4.51)	1.90 (0.82–4.36)	1.91 (0.83–4.43)	1.78 (0.76–4.17)
**1,25(OH)**_**2**_**D**						
Lowest Quintile (<31.5 pmol/l)	668	62 (9.3)	3.97 (1.74–4.18)	3.37 (1.98–5.71)	3.11 (1.82–5.29)	2.57 (1.47–4.49)
Second Lowest Quintile (31.5–49.0 pmol/l)	668	43 (6.4)	2.23 (0.93–2.43)	2.07 (1.20–3.59)	2.02 (1.16–3.51)	1.85 (1.05–3.25)
Intermediate Quintile (49.1–63.0 pmol/l/l)	668	30 (4.5)	1.49 (0.84–2.66)	1.42 (0.79–2.54)	1.38 (0.77–2.48)	1.36 (0.75–2.45)
Second Highest Quintile (63.1–81.0 pmol/l)	668	31 (4.6)	1.58 (0.89–2.80)	1.55 (0.87–2.75)	1.49 (0.84–2.65)	1.48 (0.82–2.65)
Highest Quintile (>81.0 pmol/l)	668	20 (3.0)	1.0 (reference)	1.0 (reference)	1.0 (reference)	1.0 (reference)

Model 1: adjusted for age and gender

Model 2: adjusted as in model 1 and for body mass index, redo, operation priority, and type of surgery

Model 3: adjusted as in model 2 and for left ventricular ejection fraction, NYHA function class, and EuroSCORE

Model 4: adjusted as in model 3 and for kidney function (eGFR), inflammatory process (CRP), and diabetes mellitus

In the age- and gender-adjusted model, the OR for patients in the lowest 25OHD and 1,25(OH)_2_D category was non-significantly and significantly higher, namely 1.77 (95%CI: 0.96–3.28; P = 0.071) and 3.97 (95%CI: 1.74–4.18; P<0.001), compared with the respective reference group. In the fully adjusted model, the OR for patients in the lowest 25OHD category was attenuated and did not differ significantly from the reference category. The ORs remained however significantly higher for the patients in the two lowest 1,25(OH)_2_D categories, compared with the highest 1,25(OH)_2_D category (Table 3). Results did not differ substantially if only non-diabetes patients (n = 2484) or patients ≥ 70 years (n = 1887) were included in the data analysis (S1 and S2 Tables). Similar results were also obtained in sensitivity analyses, when 25OHD levels < 50 nmol/l were considered as lowest 25OHD category or 1,25(OH)_2_D levels were divided into tertiles. In detail, the fully adjusted OR for patients in the 25OHD category < 50 nmol/l was 1.44 (95%CI: 0.78–2.67; P = 0.244), compared with the reference category. Regarding 1,25(OH)_2_D, the OR for patients in the lowest tertile was 2.06 (95%CI: 1.36–3.10; P = 0.001), compared with the highest tertile. If all cases of infection (n = 430) were considered in the statistical analysis, the ORs for the lowest 25OHD category and lowest 1,25(OH)_2_D quintile was 1.32 (95%CI: 0.87–2.00; P = 0.197) and 2.09 (95%CI: 1.46–3.02; P<0.001), compared with the respective reference category. If only patients with thoracic wound infection were compared with non-infected patients, the ORs for the lowest 25OHD category and lowest 1,25(OH)_2_D quintile was 2.47 (95%CI: 0.76–8.10; P = 0.134) and 4.12 (95%CI: 1.73–9.80; P = 0.001), compared with the respective reference category.

## Discussion

Our data support earlier findings [12] that despite antibiotics prophylaxis, postoperative infection remains a significant complication in cardiac surgical patients. To improve prophylaxis, the use of second- or third-generation cephalosporins as well as prophylaxis prolongation up to 48 h post-operatively has been recommended in cardiac surgical patients [12]. However, the broad spectrum of different pathogens and infections in our study cast doubt that this strategy would completely solve the problem of postoperative infection.

In our study, low 1,25(OH)_2_D levels were independently associated with an increased risk of a composite of clinically relevant infections. The risk was lowest in patients with circulating 1,25(OH)_2_D levels above 81.0 pmol/l. No such association was observed for circulating 25OHD levels. Thus, our data do not support a systematic review and meta-analysis of observational studies that found a significantly increased risk of infection and sepsis in critically ill patients with circulating 25OHD levels below 50 nmol/l [23]. However, it is noteworthy that the meta-analysis reported risk ratios for infection and sepsis of 1.49 (95%CI: 1.12–1.99) and 1.46 (95%CI: 1.27–1.68), respectively, which are on average very similar to the non-significantly higher OR we observed in the sensitivity analysis of our study, compared with adequate 25OHD levels. Thus, our data do not definitively rule out a higher risk of infection at insufficient or deficient 25OHD levels. With respect to 1,25(OH)_2_D, our results support data of a small earlier study in cardiac surgical patients [24]. In that investigation, lower circulating 1,25(OH)_2_D levels were significantly associated with higher risk of a composite of low cardiac output syndrome, infection, or in-hospital death. Notably, infection was the most prevalent complication (n = 4) among the 7 postoperative events that occurred.

Experimental studies provide insights into potential mechanisms of 1,25(OH)_2_D-mediated effects on the immune system: vitamin D receptors are expressed in monocytes and these cells differentiate into macrophages under the influence of 1,25(OH)_2_D [25]. Macrophages express their own 1α-hydroxylase isoenzyme which intracellularly synthesizes 1,25(OH)_2_D from its precursor 25OHD [26]. 1,25(OH)_2_D directly and indirectly regulates the expression of important antimicrobial proteins, such as cathelicidin and defensins [27,28], and of lysosomal enzymes and reactive oxygen species like nitric oxide [29]. The combination of antimicrobial peptides and oxygen species may destroy intracellular viruses, fungi, and bacteria in the autolysosomes. Notably, monocytic cathelicidin production is reduced in individuals with low 25OHD and 1,25(OH)_2_D levels [30].

Normalizing tissue 25OHD may be necessary for providing adequate amounts of substrate for local tissue production of 1,25(OH)_2_D [31]. Interestingly, in patients with low circulating 1,25(OH)_2_D levels administration of 1,25(OH)_2_D can increase 25OHD uptake in monocytes [32], suggesting that adequate circulating 1,25(OH)_2_D levels are necessary for sufficient 25OHD availability in vitamin D target cells.

Usually, 1,25(OH)_2_D synthesis is suppressed by low substrate availability, e.g. deficient 25OHD levels [15]: In children and young female adults, for instance, an increase in circulating 25OHD of 10 nmol/l was associated with an increase in circulating 1,25(OH)_2_D of approximately 5 pmol/l [33,34]. In patients of the CALCITOP study, however, the corresponding increase in circulating 1,25(OH)_2_D levels was only 0.7 pmol/l [21], whereas several clinical parameters such as kidney function, EuroSCORE (a surgical risk score), diabetes, CRP, and diuretic use were inversely correlated with circulating 1,25(OH)_2_D levels in these patients. Similarly, in heart transplant recipients kidney function and inflammatory parameters were much more predictive of circulating 1,25(OH)_2_D levels than circulating 25OHD levels [35]. These differences between healthy individuals and cardiac surgical patients may at least in part explain why vitamin D supplements have beneficial effects on upper respiratory tract infection in otherwise healthy individuals [14,15], whereas deficient 25OHD levels (as an indicator of nutritional vitamin D status) were not significantly associated with infection in the present study. Consequently, we should not be too enthusiastic to believe that in the clinical setting simple vitamin D supplementation would be able to restore all vitamin D-related derangements.

It may well be that in our fully adjusted statistical model the association between low circulating 1,25(OH)_2_D levels and postoperative infections was underestimated by the fact that adjustments were made for those parameters which are related to both clinical outcome and circulating 1,25(OH)_2_D levels. Circulating 1,25(OH)_2_D is related to eGFR, even in individuals without chronic kidney disease [36]. As mentioned before, in the patients of the CALCITOP study 1,25(OH)_2_D was also inversely interrelated with EuroScore, diabetes and CRP values [21]. Therefore, we cannot definitively rule out that our study results are biased by over-adjustment. Of note, our data indicate a stronger association of circulating 1,25(OH)_2_D with the risk of infection in models not adjusted for the aforementioned parameters.

Our study has several strengths, such as the large number of included patients, the availability of data on both 25OHD and 1,25(OH)_2_D levels, and the short follow-up period. However, some limitations also have to be addressed. First, we do not have exact data on postoperative antibiotics use in our study cohort. Second, no data on circulating postoperative 1,25(OH)_2_D levels were available in our study. Earlier investigations could demonstrate a significant transient cardiac surgery-related decline in circulating 1,25(OH)_2_D [24], which may have contributed to the increased risk of infections. Postoperative concentrations were, however, consistently lower in patients with relatively low preoperative 1,25(OH)_2_D levels than in patients with relatively high preoperative levels of this vitamin D metabolite [24], suggesting that the preoperative 1,25(OH)_2_D level is also indicative for the postoperative 1,25(OH)_2_D level of a patient. Third, no subgroup analyses regarding the association of vitamin D with gram positive bacteria, gram negative bacteria, or fungi could be performed, because many patients were simultaneously infected with two or three of these major groups of pathogens.

Finally, there is evidence for novel pathways of vitamin D_3_ metabolism initiated by CYP11A1 [37,38], indicating that vitamin D action is not only mediated by the sequence vitamin D_3_ → 25OHD_3_ → 1,25(OH)_2_D_3_, but also by other metabolites which were not analyzed in this study.

In conclusion, our data indicate an independent association of low circulating 1,25(OH)_2_D levels with the risk of postoperative infections in cardiac surgical patients, whereas 25OHD levels were not significantly related to the risk of infection. Future studies should therefore pay more attention on circulating 1,25(OH)_2_D, its regulation, and clinical relevance.

## Supporting Information

S1 Table Multivariable-adjusted odds ratio (OR) for the primary endpoint in non-diabetes patients by cutoffs of 25-Hydroxyvitamin D and 1,25-Dihydroxyvitamin D (DOCX)

S2 Table Multivariable-adjusted odds ratio (OR) for the primary endpoint in patients ≥ 70 years by cutoffs of 25-Hydroxyvitamin D and 1,25-Dihydroxyvitamin D (DOCX)
