# Supplementary material for: Circulating 25-Hydroxyvitamin D and 1,25-Dihydroxyvitamin D Concentrations and Postoperative Infections in Cardiac Surgical Patients: The CALCITOP-Study
Source: PLoS One. 2016 Jun 29;11(6):e0158532. doi: 10.1371/journal.pone.0158532 (PMC4927161; doi:10.1371/journal.pone.0158532)
Supplement: S1 Table — (DOCX) [file pone.0158532.s001.docx]

**S1 Table**: **Multivariable-adjusted odds ratio (OR) for the primary endpoint in non-diabetes patients by cutoffs of 25-Hydroxyvitamin D and 1,25-Dihydroxyvitamin D**

| Vitamin D | Primary Endpoint  N (%) | Model 1  OR (95% CI) | Model 2  OR (95% CI) | Model 3  OR (95% CI) | Model 4  OR (95% CI) |
| --- | --- | --- | --- | --- | --- |
| 25OHD  <30 nmol/l  30-49.9 nmol/l  50-74.9 nmol/l  75-100 nmol/l | 25 (4.5)  37 (4.2)  30 (4.5)  7 (2.7) | 1.77 (0.75-4.15)  1.63 (0.72-3.69)  1.76 (0.76-4.06)  1.0 (reference) | 1.97 (0.81-4.76)  1.90 (0.81-4.44)  2.00 (0.84-4.76)  1.0 (reference) | 1.80 (0.74-4.38)  1.86 (0.79-4.36)  2.05 (0.86-4.90)  1.0 (reference) | 1.84 (0.75-4.48)  1.87 (0.79-4.40)  2.00 (0.83-4.79)  1.0 (reference) |
| >100 nmol/l  1,25(OH)_2_D  <31.5 pmol/l  31.5-49.0 pmol/l  49.1-63.0 pmol/l/l  63.1-81.0 pmol/l  >81.0 pmol/l | 10 (8.3)  35 (9.5)  20 (4.2)  16 (3.1)  27 (4.1)  18 (3.1) | 3.25 (1.21-8.78)  3.05 (1.69-5.51)  1.27 (0.66-2.44)  0.94 (0.47-1.87)  1.30 (0.69-2.46)  1.0 (reference) | 2.78 (0.98-7.86)  2.47 (1.34-4.54)  1.12 (0.58-2.18)  0.88 (0.44-1.77)  1.29 (0.68-2.45)  1.0 (reference) | 2.64 (0.93-7.50)  2.28 (1.24-4.22)  1.17 (0.57-2.15)  0.87 (0.43-1.74)  1.24 (0.65-2.36)  1.0 (reference) | 2.61 (0.92-7.46)  2.13 (1.11-4.05)  1.08 (0.55-2.12)  0.89 (0.44-1.78)  1.27 (0.66-2.43)  1.0 (reference) |

Model 1: adjusted for age and gender

Model 2: adjusted as in model 1 and for body mass index, redo, operation priority, and type of surgery

Model 3: adjusted as in model 2 and for left ventricular ejection fraction, NYHA function class, and EuroSCORE

Model 4: adjusted as in model 3 and for kidney function (eGFR), inflammatory process (CRP), and diabetes mellitus
