# Supplementary material for: Circulating 25-Hydroxyvitamin D and 1,25-Dihydroxyvitamin D Concentrations and Postoperative Infections in Cardiac Surgical Patients: The CALCITOP-Study
Source: PLoS One. 2016 Jun 29;11(6):e0158532. doi: 10.1371/journal.pone.0158532 (PMC4927161; doi:10.1371/journal.pone.0158532)
Supplement: S2 Table — (DOCX) [file pone.0158532.s002.docx]

**S2 Table**: **Multivariable-adjusted odds ratio (OR) for the primary endpoint in patients ≥ 70 years by cutoffs of 25-Hydroxyvitamin D and 1,25-Dihydroxyvitamin D**

| Vitamin D | Primary Endpoint  N (%) | Model 1  OR (95% CI) | Model 2  OR (95% CI) | Model 3  OR (95% CI) | Model 4  OR (95% CI) |
| --- | --- | --- | --- | --- | --- |
| 25OHD  <30 nmol/l  30-49.9 nmol/l  50-74.9 nmol/l  75-100 nmol/l | 33 (6.9)  35 (5.2)  26 (5.7)  8 (4.4) | 1.71 (0.77-3.79)  1.22 (0.56-2.68)  1.31 (0.58-2.96)  1.0 (reference) | 1.93 (0.85-4.38)  1.39 (0.62-3.11)  1.56 (0.68-3.59)  1.0 (reference) | 1.92 (0.84-4.41)  1.51 (0.67-3.43)  1.83 (0.78-4.26)  1.0 (reference) | 1.75 (0.76-4.03)  1.40 (0.62-3.19)  1.69 (0.72-3.96)  1.0 (reference) |
| >100 nmol/l  1,25(OH)_2_D  <31.5 pmol/l  31.5-49.0 pmol/l  49.1-63.0 pmol/l/l  63.1-81.0 pmol/l  >81.0 pmol/l | 10 (10.6)  39 (10.1)  25 (5.9)  23 (5.7)  18 (5.2)  9 (2.8) | 2.63 (1.00-6.91)  3.89 (1.85-8.16)  2.13 (0.98-4.63)  2.09 (0.95-4.58)  1.88 (0.83-4.25)  1.0 (reference) | 2.23 (0.81-6.14)  3.51 (1.66-7.42)  2.17 (0.99-4.75)  2.08 (0.94-4.59)  1.92 (0.84-4.36)  1.0 (reference) | 2.46 (0.88-6.84)  3.08 (1.44-6.59)  2.04 (0.93-4.49)  1.99 (0.90-4.42)  1.79 (0.79-4.09)  1.0 (reference) | 2.01 (0.71-5.68)  2.63 (1.19-5.80)  1.92 (0.86-4.30)  2.03 (0.91-4.56)  1.82 (0.79-4.19)  1.0 (reference) |

Model 1: adjusted for gender

Model 2: adjusted as in model 1 and for body mass index, redo, operation priority, and type of surgery

Model 3: adjusted as in model 2 and for left ventricular ejection fraction, NYHA function class, and EuroSCORE

Model 4: adjusted as in model 3 and for kidney function (eGFR), inflammatory process (CRP), and diabetes mellitus
